# Clinical and Serological Profiles in Cryoglobulinemia: Analysis of Isotypes and Etiologies

**DOI:** 10.3390/jcm13206069

**Published:** 2024-10-11

**Authors:** Helena Codes-Méndez, Sicylle Jeria, Hye-Sang Park, Patricia Moya, Berta Magallares-López, Elisabeth Moltó, Yolanda Álvaro, Anais Mariscal, Esther Moga, Jose Luis Tandaipan, César Díaz-Torne, Ana Laiz, Luis Sainz, Ivan Castellví, Hector Corominas

**Affiliations:** 1 Department of Rheumatology, Hospital de la Santa Creu i Sant Pau, 08025 Barcelona, Spain; hcodes@santpau.cat (H.C.-M.); sjeria@santpau.cat (S.J.); hsang@santpau.cat (H.-S.P.); pmoyaa@santpau.cat (P.M.); bmagallares@santpau.cat (B.M.-L.); jtandaipan@santpau.cat (J.L.T.); cdiazt@santpau.cat (C.D.-T.); alaiz@santpau.cat (A.L.); lsainz@santpau.cat (L.S.); icastellvi@santpau.cat (I.C.); 2 Institut de Recerca Sant Pau (IR SANT PAU), Sant Quintí 77-79, 08041 Barcelona, Spain; 3 Multi-Organ Damage and Rheumatology Group, Sant Pau Biomedical Research Institute (IIB Sant Pau), 08025 Barcelona, Spain; 4 Medicine Faculty, Universitat Autònoma de Barcelona (UAB), 08193 Cerdanyola del Vallès, Spain; 5 Immunology Department, Hospital de la Santa Creu i Sant Pau, 08025 Barcelona, Spain; emolto@santpau.cat (E.M.); yalvaro@santpau.cat (Y.Á.); amariscal@santpau.cat (A.M.); emoga@santpau.cat (E.M.)

**Keywords:** cryoglobulinemia, vasculitis, hepatitis C, rheumatic disease, hematological disease

## Abstract

**Objectives**: Cryoglobulinemia (CG) is marked by abnormal immunoglobulins (Ig) in serum, precipitating at temperatures below 37 °C. Current classification categorizes CG into three subtypes (types I, II, and III) based on Ig clonality. The features distinguishing patients with CG based on their etiology remain unidentified. Aiming to characterize clinical and serological profiles of CG individuals, we conducted an observational analysis of a large cohort of patients and compared their characteristics based on underlying causes: hepatovirus (HV) infections, rheumatic diseases (RD), hematological disorders, and unidentified etiology (essential CG). **Methods**: We analyzed 252 cryoglobulin-positive serum samples from 182 patients and classified these into the four etiological groups. A separate sub-analysis was carried out for 10 patients meeting criteria for multiple diseases. We collected demographic, clinical, and laboratory data: CG characterization, complement (C3 and C4) levels, antinuclear antibodies (ANA), and rheumatoid factor (RF). Kruskal–Wallis and Wilcoxon–Mann–Whitney *U-*tests were used for comparisons. **Results**: Most patients (93.3%) had mixed cryoglobulinemia (types II + III), with 6.7% having type I. HV infection, predominantly hepatitis C, was the main (52.9%) associated condition within the cohort, followed by rheumatic (27.3%) and hematological (9.8%) disorders. In our cohort, ANA were frequent (45.3%) and often associated with RF positivity (43.6%) and decreased complement levels (C3: 42.4%, C4: 32.5%). Essential CG and CG associated with RD had a higher prevalence of cutaneous manifestations (*p* < 0.01) and renal involvement (*p* = 0.017). Hematological disorder-related CG showed higher cryoglobulin and RF concentrations (*p* < 0.01), despite milder symptoms. **Conclusions**: Our study underscores a mixed prevalence of CG across disease subgroups, with hepatitis-C virus as the primary factor, followed by rheumatic and hematological disorders. Four clinical and serological profiles of CG were identified based on their etiologies.

## 1. Introduction

Cryoglobulinemia (CG) is a rare phenomenon characterized by the persistent presence of abnormal immunoglobulins (Igs) in the bloodstream. These abnormal Igs form precipitates in vitro at temperatures below 37 °C, but dissolve again upon rewarming [1,2]. If the amount of cryoglobulin is high, precipitation may even occur at room temperature [3]. The precise biochemical mechanisms that precipitate cryoglobulin upon cold exposure have not been completely elucidated [4,5]. Complement activation plays a significant role by attaching to endothelial cell receptors, thus aiding in the deposition of immune complexes and causing subsequent inflammation in small- and medium-sized vessels [6]. The clinical signs of disease, caused by the in vivo precipitation of these complexes in small- and medium-sized blood vessels [7], define the term cryoglobulinemic syndrome or cryoglobulinemic vasculitis.

The main mechanism contributing to CG is aberrant autoantibody production by plasma cells and B-cell clonal expansion. This process can be facilitated by lymphoproliferative disorders, persistent immune stimulation due to chronic infections, or autoimmune diseases [8,9]. Consequently, CG is recognized as a condition encompassing a broad spectrum of potential etiologies, diverse pathogenic mechanisms, a wide array of phenotypic manifestations, and a pronounced interplay between infection, autoimmunity, and neoplastic processes.

Globally, cryoglobulinemic syndrome is a rare disease, with a prevalence of <5 in 100,000 individuals. It primarily affects females, with a ratio of 3-to-1, and age at onset is generally within the range of 42 to 52 years [10]. The disorder is often recognized by the classic triad of palpable purpura, arthralgia, and asthenia or weakness [1], present in as many as 80% of patients at disease onset [8,9]. However, it may manifest with a broad spectrum of symptoms, affecting the skin, joints, nerves, and kidneys [8,11], and even the gastrointestinal and central nervous system in rare cases. However, not all cases of CG exhibit noticeable symptoms, as it may be incidentally detected during routine laboratory assessments [11]. When we consider and evaluate the various causes of CG, it becomes evident that the various etiologies are associated with distinct clinical presentations.

Serum biomarkers play a crucial role in the diagnosis, prognosis, and potential treatment adjustments for CG. Notably, rheumatoid factor (RF), antinuclear antibodies (ANA), and levels of classical complement pathway factors (C1, C2, C4) have shown to be particularly valuable in assessing the underlying etiologies and the prognosis of CG syndrome and vasculitis [12].

In 1974, Brouet et al. [13] proposed a classification system for CG based on chemical and immunological findings. They delineated three distinct subtypes: subtype I CG is characterized by the presence of a single monoclonal immunoglobin, commonly of the IgG or IgM isotypes, and occasionally IgA. Types II and III CG, often referred to as mixed cryoglobulinemia (MCG), are characterized by a combination of polyclonal IgG along with monoclonal IgM (type II) or polyclonal IgM (type III) with RF activity.

Type I CG is usually a serological finding observed during the course of hematological disorders such as monoclonal gammopathy of undetermined significance (MGUS), malignancies of B-cell lineage, mainly multiple myeloma (MM), and Waldenström macroglobulinemia (WM) [10]. Conversely, the detection of types II and III MCG is the main laboratory hallmark of cryoglobulinemic vasculitis. Type II CG is predominantly associated with hepatitis-C virus (HCV) infection, although additional causative factors such as other virus infections [14,15], rheumatic diseases (RD), and lymphoproliferative disorders are also plausible etiologies. Type III CG arises in the context of RD or secondary to infections, particularly HCV. Overall, MCG results from a B-cell lymphoproliferative process in the context of persistent immune activation triggered by chronic infections, systemic autoimmune diseases, or an unknown origin [16,17], with approximately 10% of patients having no identifiable cause, in which case the disorder is recognized as essential cryoglobulinemia [8].

This proposal based on CG type remains widely used [18,19,20,21,22]. Another prevalent approach involves categorizing the presence of CG according to its association with HCV infection [23,24,25,26]. Our decision to undertake this study originated from our routine clinical practice, where we systematically analyze CG within the context of its underlying etiological causes.

Hence, the primary objective of our study was to characterize the clinical and serological profiles of individuals with CG and elucidate variations based on their underlying etiologies, including hepatovirus (HV) infections, RD, hematological disorders, and essential CG.

## 2. Materials and Methods

### 2.1. Study Population

A large monocentric cross-sectional study was conducted at a rheumatology outpatient clinic within a tertiary referral teaching hospital in Barcelona, assessing a population of approximately 450,000 individuals.

All instances of positive cryoglobulin determinations in serum, as part of the standard diagnostic and/or follow-up evaluations, were consecutively collected from November 2019 to November 2021. These samples were categorized into four groups based on their etiological origins: (1) RD, encompassing patients with rheumatoid arthritis (RA), systemic lupus erythematosus (SLE), Sjögren’s syndrome (SjS), and systemic sclerosis (SSc); (2) hepatitis viruses, including patients diagnosed with either HCV, hepatitis-B virus (HBV), or both; (3) hematological diseases, with patients whose CG could be attributed to hematologic disorders; and (4) essential CG, comprising patients with CG where rheumatic, infectious or hematologic disease had been ruled out as the underlying cause. A cohort of 182 patients was included in this study, with some individuals contributing more than one sample, thus yielding a total of 252 serum samples. All these samples presented cryoglobulinemia levels above 8 mg/dL. Samples were collected as part of routine clinical practice, representing a collective of patients within a real-life clinical setting.

Some patients, particularly in the RD group, had received immunosuppressive therapy prior to CG evaluation, which might have influenced their CG levels. Patients who fulfilled the diagnostic or classification criteria for multiple disease categories within the aforementioned four groups, potentially impacting result interpretation, underwent separate analysis. Their data have been included in a distinct section of the results.

Patients with concomitant conditions such as cancer or chronic infectious diseases (including Epstein–Barr virus, varicella-zoster virus, cytomegalovirus, parvovirus B19, and measles) were excluded to prevent interference with the interpretation of clinical and biomarker data.

The study was approved by the institutional ethics committee of Hospital de la Santa Creu i Sant Pau (code: IIBSP-BIR-2017-07), and performed in accordance with the Helsinki Declaration.

### 2.2. Data Collection and Variables

Patient data were collected from the electronic healthcare records of the hospital and included demographic variables, clinical manifestations, and laboratory parameters. The demographic information gathered included gender, birthdate, and age at diagnosis of CG.

Assessment of clinical features included the following: (i) joint involvement: non-erosive arthritis characterized by tenderness or swelling affecting two or more peripheral joints; (ii) cutaneous involvement: Raynaud’s syndrome, purpura with positive skin biopsies showing leukocytoclastic vasculitis, acral cyanosis, acral ischemia affecting areas such as the nose, ears, fingers, leg, or toes, and ulcers affecting the legs or fingers; (iii) neurological involvement: peripheral neuropathy confirmed by electromyography or nerve biopsy; and (iv) renal involvement: chronic kidney disease exceeding stage 3 (GFR ≤ 60 mL/min/173 m^2^) and/or glomerular nephropathy, diagnosed by renal biopsy.

The laboratory evaluation consisted of quantification of protein cryoprecipitate and evaluation of Ig (G, A, and M) presence through immunodiffusion. Biomarkers analyzed were the titer and pattern of ANA, complement (C3 and C4), RF, beta-2 microglobulin (B2M) levels, C-reactive protein (CRP), and erythrocyte sedimentation rate (ESR).

Cryoglobulinemic vasculitis was classified based on the validated criteria established by the Italian Group for the Study of Cryoglobulinemia in 2014 [27,28]. Diagnosis required the presence of CG in the serum with at least two of the following clinical features: (1) purpura, (2) weakness, (3) arthralgia, or (4) peripheral neuropathy. Additionally, at least two of the following laboratory findings were necessary: (1) reduced serum C4, (2) positive RF, and/or (3) positive serum monoclonal (M) component. Histopathological or laboratory evidence of vasculitis was also used to further confirm the diagnosis. These criteria were applied to ensure accurate diagnosis and consistent classification of cryoglobulinemic vasculitis within our cohort.

### 2.3. Laboratory Investigation of Cryoglobulinemia and Serum Biomarkers

Blood samples were obtained one to seven days before the clinical evaluation. To determine protein cryoprecipitate, stringent conditions were implemented to mitigate the risk of false-negative results arising from improper pre-analytical procedures. All samples were collected using preheated syringes and tubes. Upon arrival at the laboratory, samples were incubated at 37 °C 1 h prior to centrifugation at room temperature and serum collection. The obtained serum was separated into two aliquots and then stored with 0.1 g/L sodium azide for 4–7 days at 2–8 °C before cryoglobulin evaluation. One of the two aliquots from each sample was processed at 37 °C and the other at 4 °C. The aliquot to be processed at 37 °C was first incubated at 37 °C for 1 h. Samples were washed three times at 37 °C or 4 °C through centrifugation (10,000× *g* 3 min) and finally incubated at 37 °C 1 h before spectrophotometric protein quantification. Protein cryoprecipitate was calculated according to the following formula: 260 × (absorbance of the 4 °C aliquot—absorbance of the 37 °C aliquot). In samples with a protein cryoprecipitate > 8 mg/dL, the cryoprecipitate was submitted to immunodiffusion to qualitatively analyze the presence of Ig (G, A, and M) and complement (C3 and C4).

To minimize the risk of false-positive results in cryoglobulin diagnosis, our protocol includes a two-step process: an initial screening step to measure proteins in the cryoprecipitate, followed by a typing step for samples that test positive during screening. This approach enhances diagnostic accuracy and reduces the likelihood of false positives.

Patients were categorized as type I CG when only one Ig isotype was detected in the immunodiffusion, and as MCG (types II and III) when more than one Ig isotype was detected.

As part of routine clinical assessment, we analyzed various inflammatory markers, namely CRP, ESR, and B2M. Upper normal limits were defined at 5 mg/L for CRP, 15 mm/h for ESR, and 1.80 mg/L for B2M. ANA levels were detected via indirect immunofluorescence on HEp-2 cells, with a titer cut-off of 1:80. Additionally, complement C3 and C4 levels were measured with a lower threshold of 85 mg/dL for C3 and 15 mg/dL for C4. RF positivity was assessed using a cut-off of 20 IU/mL.

### 2.4. Statistical Analysis

Descriptive statistics are presented as absolute frequencies, including both median with interquartile range (IQR) and mean with standard deviation (SD), when appropriate. The Shapiro–Wilk test and box plot were used to check distribution of the variables.

To address the primary objective of this study, we performed a comparative analysis between groups using the multivariate Kruskal–Wallis test for continuous variables and Fisher’s exact test for qualitative variables. Statistical significance was established at a *p*-value of <0.05. Data analysis was performed using Stata 16.0.

## 3. Results

### 3.1. Baseline Clinical Characteristics and the Distribution of Biomarkers according to Disease Subgroups

Of the initial 182 patients under review, data analysis included 172 individuals. Mean age at CG diagnosis in our cohort was 59.7 years (±14.5), and 117 patients (68%) were female. Demographic and clinical characteristics are summarized in Table 1, and detailed laboratory findings are presented in Table 2.

We determined the CG subtype in 160 of 172 patients, representing 93% of the total cohort. We were unable to determine the CG subtype in 12 patients. Among the 160, 11 patients (6.8%) were type I and all exhibited associations with hematological disorders. Among these type I cases, the IgM subtype was predominant, being present in eight patients. Of 172 patients, 149 (93.1%) exhibited MCG, with the presence of both IgG and IgM being the most prevalent combination (in 110 of the 149 patients).

These patients were distributed across the three etiological groups as follows: 72 from the HV group, 26 from the RD group, and 12 from the essential CG group. Less common CG subtypes were also identified, and included combinations involving IgG, IgM and IgA, IgG and IgA, and IgM and IgA. CG subtypes within our cohort are summarized in Table 3.

Monoclonal gammopathy was studied in 84 patients, reflecting robust data availability. Further analysis revealed that 20 out of 84 (23.8%) cases exhibited gammopathies, with IgM being the predominant component in 55% of cases, primarily within the HCV-associated group.

A total of 20 patients underwent renal biopsy, of whom 12 were confirmed to have glomerulonephritis associated with cryoglobulinemic vasculitis. The remaining biopsies indicated glomerulonephritis related to underlying diseases and were excluded from the renal involvement group attributed to cryoglobulinemic vasculitis.

### 3.2. Hepatotropic Viruses’ Infection

Among groups, hepatotropic viral infection was the most prevalent etiology of CG in our cohort, affecting 91 (82.7%) patients within the MCG subgroup. Within this group, 90 patients were HCV-positive, one had HBV infection, and one had concurrent HBV and HCV. Additionally, one patient was co-infected with HIV along with HBV and HCV. The predominant symptoms in this group of patients included skin purpura (9.9%), arthritis (8.8%), and peripheral neuropathy (9.9%), whereas they exhibited a lower rate of renal involvement than those in the other groups, with only three (3.3%) patients exhibiting glomerulonephritis and nine (9.9%) patients having chronic kidney disease stage ≥ 3. ANA were detected in 27 (48.2%) patients, with high titers (≥1:640) observed in only three (11%) of them. Among the 27 ANA-positive patients, the predominant patterns were the speckled pattern in 15 (55.5%) and the cytoplasmic pattern in seven (25.9%). Moreover, this subgroup of patients exhibited a significant reduction in complement C3 and C4 levels (*p* = 0.0001), mostly in ANA-positive patients. RF positivity was found in 44 (51.2%) individuals, although no severe clinical manifestations were associated with its presence.

### 3.3. Rheumatic Diseases

We found 47 patients with RD, of whom 32 (68.1%) had primary SjS, eight (17%) had SLE, three (6.4%) had RA, and four (8.5%) had SSc. Remarkably, they had a significantly higher prevalence of Raynaud’s syndrome and non-erosive arthritis compared with the other subgroups (*p* = 0.0001), along with cold-induced acrocyanosis (*p* = 0.0033). Peripheral neuropathy was recognized in 10 (21.3%) patients, with five (10.6%) patients having mixed polyneuropathy and five (10.6%) having multiple mononeuritis. Renal involvement with chronic kidney disease stage ≥ 3 was observed in seven (14.9%) patients, with the majority (five out of the seven) showing signs of glomerulonephritis. Among these cases, two SjS patients had interstitial nephritis, one SjS had membranoproliferative GN, and two SLE patients had segmental and focal GN. Furthermore, 80.4% of patients tested positive for ANA, with high titers in a significant proportion (48.7%). The predominant ANA patterns observed were the speckled pattern in 35.1% of cases and the mixed homogeneous pattern with the speckled pattern in 24.3%.

### 3.4. Hematological Diseases

Hematological diseases were present in 17 (9.9%) patients, 11 being cases of MGUS, two of T-cell lymphoma, two WM, one MM, and one chronic lymphocytic leukemia. Among these 17 patients, 12 (70.5%) had a monoclonal component, with IgM kappa being the predominant subtype in eight cases (72.7%). This group displayed the highest mean serum protein cryoprecipitate levels (range: 292.4 mg/dL ±546.2) (*p* = 0.0001) despite the absence of significant clinical manifestations. ANA positivity was less common than in the other subgroups, and none of the patients showed a decrease in C4 (Table 2).

### 3.5. Essential Cryoglobulinemia

The essential CG group comprised 9.8% of the sample and was predominantly characterized by MCG in 15 out of 17 patients. Regarding clinical manifestations, this fourth subgroup had the highest occurrence of skin and renal involvement. Cutaneous symptoms were observed in nine patients (52.9%) (*p* = 0.0001), most of them presenting with purpura, and renal failure with chronic kidney disease stage ≥ 3 was noted in seven (41.2%) patients (*p* = 0.0170). ANA positivity was evaluated in 16 patients, revealing a positive result in nine (56.3%) cases, with a predominant speckled pattern observed in seven (43.8%) of them. Laboratory results demonstrated markedly elevated levels of RF in this particular group (*p* = 0.00010).

### 3.6. Analysis of 10 Patients Meeting Diagnostic Criteria across Multiple Disease Subgroups

Ten patients fulfilled diagnostic criteria for more than one disease category and were therefore excluded from the main statistical analysis. These ten individuals presented with various combinations of conditions: SjS with HCV in three cases, SjS and MGUS in one case, RA and MGUS in one case, and HCV and MGUS in one case. They had also exhibited other pathologies, such as antiphospholipid syndrome (APLS) in two cases, multiple sclerosis in one case, and inflammatory myopathy in one case. Among the 10 individuals, two had Raynaud’s phenomenon (one with SjS and HCV, and one with inflammatory myopathy) and two had non-erosive arthritis (one with APLS, and one with SjS and HCV). Laboratory findings in this group of 10 patients revealed MCG with IgG-IgM isotypes and ANA-positive, but with titers below 1/320 in all cases. The two patients with both SjS and HCV displayed higher protein cryoprecipitate than that of the remaining eight patients (mean of 108 mg/dL vs. 9 mg/dL, respectively), together with higher levels of RF (mean of 440 IU/mL). However, only one of these two patients showed C4 consumption (Table 2).

## 4. Discussion

Cryoglobulinemic syndrome is a multifaceted and often underdiagnosed clinical entity, emerging as a shared manifestation across various underlying conditions, including autoimmune, infectious, and hematological disorders. We performed a comprehensive analysis of clinical and laboratory findings in patients with CG, and compared their characteristics based on their underlying etiologies.

First, consistent with prior literature [29], in our cohort we observed that MCG constituted the predominant subtype, being present in the majority of patients (93.3%). MCG was predominantly observed in patients with hepatotropic virus infection, though it was also identified across other etiological groups. Our findings corroborate and extend upon prior studies, reinforcing the pivotal role of HCV in the majority of MCG cases. Consistent with historical trends, HCV was the primary driver of MCG, accounting for 82.7% of cases. Patients with HCV-related CG vasculitis typically presented a decrease in complement, particularly C4, in accordance with prior reports [26,30]. This suggests that continuous exposure to HCV antigens stimulates the immune system, leading to the deposition of immune complexes and in situ complement activation [31]. Although the mechanisms leading to B-cell activation and expansion are still unknown, these B-lymphocytes are dependent on HCV antigens, as CG often disappears when HCV is effectively treated [24,32,33] and it is known that patients with HCV-related CG vasculitis face a substantially elevated risk: a 35-fold increase of developing B-cell non-Hodgkin lymphoma than the general population [7,34,35].

Second, our findings underscore the significant role of RD in CG syndrome, particularly in MCG, with a notable 58% prevalence of RD in our CG cohort. This exceeds the previously reported 30% in non-infectious CG cases [18,19], potentially reflecting enhanced detection of previously underdiagnosed conditions such as SjS. In line with prior studies [36,37,38,39,40], SjS was the predominant entity in our RD subgroup, identified in 68% (32 out of 47) of RD patients. Additionally, we identified eight patients with SLE (17% of the RD group), three with RA (6.3%), and four with SSc (8.5%), which is consistent with the existing literature [16,19,38,39,40]. The established association between CG and RD, particularly with SjS and SLE, is important, even though it is less commonly reported in other RDs [17,41,42]. Our findings contribute to the growing evidence supporting the necessity of CG evaluation in these patients. Moreover, the increased risk of lymphoproliferative disorders and mortality in patients with HCV-unrelated CG vasculitis, as documented by Terrier in 2015 [18], emphasizes the need for vigilant management and early intervention. Our study also highlights the association between hematological disorders and type I CG, primarily characterized by the IgM subtype. This observation is consistent with earlier extensive studies, including the comprehensive analysis conducted by Kolopp-Sarda involving 1675 CG patients [30]. Additionally, we observed that these patients display significantly higher levels of serum cryoglobulins than individuals with MCG. This finding builds upon evidence in Roccatello’s report in 2018 [42]. Moreover, it has been previously demonstrated that these cases may occasionally be associated with hyperviscosity syndrome, albeit with a low incidence, ranging from 0 to 5% [14]. This syndrome typically manifests as the triad of mucosal bleeding, visual changes and neurological symptoms, and cold-induced acral necrosis, as reported by Ramos-Casals et al. [6] and other earlier studies [11,16]. However, none of the patients in our cohort manifested classic signs of hyperviscosity syndrome. Furthermore, we did not observe a correlation between the symptoms of cryoglobulinemic vasculitis and the protein cryoprecipitate concentration, consistent with previous reports. These findings reinforce the concept that type I CG is primarily a serological finding abnormality rather than a distinct disease entity [8,14,42].

Our study identified a substantial proportion (22.6%) of patients presenting with vasomotor and skin-related manifestations, irrespective of etiology. This underscores the critical role of CG testing in everyday clinical practice for patients with unexplained skin abnormalities, even in the absence of a clear underlying etiology. Given their expertise in skin manifestations, dermatologists are often the first healthcare providers to evaluate skin changes. However, the heterogeneity in clinical presentation is a hallmark of CG, highlighting the necessity for CG evaluation across various specialties, including nephrologists, infectious disease specialists, hematologists, and neurologists, as early recognition of CG is essential. It is worth noting that despite the high prevalence of hepatotropic virus infection within our cohort, cutaneous manifestations were relatively less frequent in this subgroup, further emphasizing the heterogeneity of clinical presentations of CG.

Renal involvement and glomerulonephritis were observed in patients from various subgroups in our cohort. As previously reported, the presence of GN, especially in the context of elevated cryoglobulin levels, should prompt clinicians to consider CG as a potential underlying cause of renal involvement [43,44]. Early recognition of renal involvement and initiation of appropriate treatment strategies are paramount to preventing further renal deterioration and complications.

Within our cohort, and in line with earlier research findings, the lowest mean protein cryoprecipitate concentration was observed in the RD group, while this group simultaneously exhibited the highest occurrence of CG symptoms [14]. Furthermore, we identified the most significant complement consumption in the HV group. Laboratory findings are crucial in both the diagnosis and monitoring of CG vasculitis. The presence of cryoglobulin in two independent analyses serves as a fundamental diagnostic criterion, while the levels of cryoglobulin and other surrogate markers such as complement levels and RF are key indicators for disease monitoring [27,38,45]. As previously documented, high serum cryoglobulin concentrations can characterize an oligosymptomatic or asymptomatic disease course, whereas low cryoglobulin values and/or a reduction in complement levels typically correlate with severe, active cryoglobulinemic syndrome. Our study revealed positivity for ANA across all etiological groups, predominantly within the RD group, characterized by a markedly higher prevalence of positivity and elevated titers exceeding 1:640, with a higher proportion of high titers (>1:640). In our investigation, we observed positivity for ANA antibodies across all etiological groups, predominantly within the RD cohort, characterized by a markedly higher prevalence of positivity and elevated titers exceeding 1:640.

The main limitation of our study is that the analysis of Ig precipitates did not differentiate precisely between type II and type III CG. Additionally, the retrospective design hindered the uniform analysis of specific laboratory parameters across the entire cohort. Another significant limitation is that some patients, particularly those in the RD group, had received immunosuppressive therapy prior to CG evaluation, which might have influenced CG levels. However, despite these limitations, our findings remain consistent with prior research [5,17,27].

The main strength of this study lies in the significance of our cohort, a large number of patients with CG across various etiologies. In contrast with the prevailing focus on HCV patients in much CG research to date, exemplified by the large study from Retamozo and Díaz-Lagares [46], research comprising all etiologies of CG is limited [20,34,47,48]. In this context, our research stands out for its inclusive approach, comprehensively including patients with CG stemming from diverse causes. Additionally, our findings hold particular relevance in the context of the declining prevalence of active HCV infection, attributable to the emergence of targeted therapies against the virus. This decline in HCV prevalence has consequently led to a significant reduction in research on CG in recent years, further underscoring the significance of our study in addressing this gap. Moreover, our study stands out for obtaining an extensive array of clinical and serological variables pertinent to CG patients.

Future research should aim to explore the underlying mechanisms driving the common immunological features observed in CG patients and their potential clinical implications. Longitudinal studies and larger multicenter cohorts will be crucial for a more comprehensive understanding of CG subtypes and their associations with various underlying diseases.

To summarize, this observational study provides valuable insights into the clinical heterogeneity and immunological features of patients with CG. Our findings underscore the importance of recognizing the diverse clinical manifestations and associated diseases within the CG spectrum. Identification of clinical and laboratory features suggestive of CG, regardless of the underlying etiology, is imperative. By elucidating the evolving landscape of CG and its associated presentations, aiding in early CG detection, we aim to guide clinical practice and facilitate targeted interventions, as necessary, to optimize patient outcomes. As a result, it is advisable to routinely screen and monitor CG from its onset, especially in individuals with MCG and either HCV infection or Sjögren’s syndrome, to prevent potential complications, such as the activation of monoclonal B-cell clones and the consequent risk of lymphoma. Further research, including larger-scale studies, is warranted to refine diagnostic criteria and therapeutic approaches for CG across diverse patient populations.

## Figures and Tables

**Table 1:** Demographic and clinical characteristics of the 172 patients.

	Rheumatic Diseases (*n* = 47)	Hepatotropic Viruses (*n* = 91)	Hematological Diseases (*n* = 17)	Essential CG (*n* = 17)	
Gender, n (%) (M/F)	5/42 (10.6/89.4)	34/57 (37.4/62.6)	10/7 (58.8/41.2)	6/11 (35.3/64.7)	
Age at CG diagnosis, years, (±SD)	60.6 (±14.0)	59.6 (±13.1)	61.1 (±16.6)	56.3 (±20.8)	
Vasculitis CG criteria *, *n* (%)	27 (57.4)	61 (67.0)	2 (11.8)	8 (47.0)	
Clinical Characteristics					
Skin, *n* (%)	18 (38.3)	10 (11.0)	2 (11.8)	9 (52.9)	*p* = 0.0001
○ Raynaud, *n* (%)	14 (29.8)	0 (0)	1 (5.9)	3 (17.6)	*p* = 0.0001
○ Purpura, *n* (%)	6 (12.8)	9 (9.9)	2 (11.8)	6 (35.3)	*p* = 0.0458
○ Cold acrocyanosis, *n* (%)	6 (12.8)	0 (0)	0 (0)	1 (5.9)	*p* = 0.0033
○ Acral ischemia, *n* (%)	1 (2.3)	0 (0)	0 (0)	1 (5.9)	*p* = 0.1780
○ Ulcers, *n* (%)	3 (6.4)	2 (2.2)	0 (0)	2 (11.8)	*p* = 0.1924
Peripheral neuropathy, *n* (%)	10 (21.3)	9 (9.9)	1 (5.9)	4 (23.5)	*p* = 0.1363
Non-erosive arthritis, *n* (%)	22 (46.8)	8 (8.8)	1 (5.9)	4 (23.5)	*p* = 0.0001
Glomerulonephritis, *n* (%)	5 (10.6)	3 (3.3)	1 (5.9)	3 (17.6)	*p* = 0.1192
Weakness/Asthenia, *n* (%)	32 (68.1)	43 (47.2)	11 (64.7)	12 (70.5)	*p* = 0.1589

CG: Cryoglobulinemia; * Vasculitis CG classification criteria [27,28].

**Table 2:** Laboratory, biochemical data, and cryoglobulinemia characteristics of patients.

	Rheumatic Diseases (*n* = 47)	Hepatotropic Viruses (*n* = 91)	Hematological Diseases (*n* = 17)	Essential CG (*n* = 17)	
Cryoglobulinemia Characteristics					
Protein cryoprecipitate (mg/dL), $\bar{x}$ (±SD) Isotype Ig, *n* (%)	26.7 (±63.2) G + M = 26 (55.3)	65.8 (±256.5) G + M = 72 (79.1)	292.4 (±546.2) M = 8 (47.1)	47.59 (±79.1) G + M = 12(70.6)	*p* = 0.0001 *p* = 0.0067
Biochemical and Immunological Features					
CKD ≥ 3 stage, *n* (%)	7 (14.9)	9 (9.9)	3 (17.6)	7 (41.2)	*p* = 0.0170
B2M (≥1.8 mg/L), *n* (%)	7/40 (17.5)	1/5 (20.0)	3/12 (25.0)	0 (0)	*p* = 0.4489
CRP (mg/L) p50	10.3 (±26.2)	3.9 (±3.0)	13.4 (±18.3)	8.5 (±12.0)	*p* = 0.4749
ESR (mm/h) p50	40.0 (±28.5)	20.3 (±20.2)	35.4 (±35.1)	24.5 (±25.0)	*p* = 0.0003
ANA+, *n* (%)	37/46 (80.4)	27/56 (48.2)	5/13 (38.5)	9/16 (56.3)	*p* = 0.0032
Titers (≥1:640)	18 (48.7)	3 (11.1)	1 (20)	2(22.2)	*p* = 0.0116
RF + (>20 IU/mL), *n* (%)	19/46 (41.3)	44/86 (51.2)	5/11 (45.5)	7/17 (41.2)	*p* = 0.0945
- $\bar{x}$ (±SD)	90.6 (±175.9)	161.0 (±219.5)	94.8 (±135.6)	284.5 (±619.3)	N/A
Low C3 (<85 mg/dL), *n* (%)	20 (42.6)	47 (51.6)	3 (17.6)	3 (17.6)	*p* = 0.1355
- $\bar{x}$ (±SD)	90.1 (±28.6)	68.5 (±10.8)	99.1 (±29.0)	114.8 (±12.7)	N/A
Low C4 (<12 mg/dL), *n* (%)	17 (36.2)	36 (39.6)	0 (0)	3 (17.6)	*p* = 0.0252
- $\bar{x}$ (±SD)	15.6 (±9.0)	7.6 (±3.5)	20.4 (±7.4)	21.1 (±9.5)	N/A

Detailed laboratory findings of the 172 patients of our cohort. Ig, immunoglobulin; CKD, chronic kidney disease; B2M, beta-2 microglobulin; CRP, C-reactive protein; ESR, erythrocyte sedimentation rate; ANA, antinuclear antibodies; RF, rheumatoid factor; C3, complement C3; C4, complement C4; N/A, not applicable.

**Table 3:** Cryoglobulin subtypes within our cohort, according to Brouet et al. classification system.

Cryoglobulin Pattern	Immunoglobulin Isotype(s)	Number of Patients (*n* = 160)
Type I (Monoclonal)		11 (6.8%)
	IgG IgM	3/11 (27.3%) 8/11 (72.7%)
Types II and III—Mixed CG (Polyclonal)		149 (93.1%)
	IgG + IgM IgG + IgA IgM + IgA IgG + IgM + IgA	110/149 (73.8%) 12/149 (8%) 6/149 (4%) 21/149 (14.1%)

## Data Availability

The datasets analyzed for this study are available on request from the corresponding author. The data is not publicly available as it contains clinical and personal information.
